# Dr. Ahmad Madani (1946–2024), Eminent Medical Teacher at the Shiraz School of Medicine

**DOI:** 10.34172/aim.31286

**Published:** 2024-08-01

**Authors:** Mohammad Hossein Azizi

**Affiliations:** ^1^Academy of Medical Sciences of the I.R. of Iran, Tehran, Iran

**With deep sorrow, Dr. Ahmad Madani, professor of neonatology at Shiraz University of Medical Sciences passed away on April 15, 2024 due to pancreatic cancer. His demise is a great loss for his family, neonatologists and Shiraz medical graduates. However, his memory will remain alive in their minds. Dr. Madani was a distinguished physician who loved kindness, beauty and truth**.

 Ahmad Madani was born in Isfahan on February 20, 1946. After primary school education, he passed his high school training in Alborz high school in Tehran. He enrolled at Isfahan School of Medicine and obtained his MD degree in 1972.^1^ In due course, in 1977, he started his pediatrics residency program at Shiraz Medical School. At the same time, he completed two additional short courses in neonatal medicine in England, and after completing the fellowship of neonatology under the supervision of Professor Mohammad Reza Sedaghatian in Shiraz, he joined the neonatal departments affiliated to the Shiraz University of Medical Sciences. In 1984, after the departure of Professor Sedaghatian abroad, Dr. Madani was initially appointed as supervisor and later, the head of the neonatal departments of Shiraz University of Medical Sciences and held this position until the end of 2004, when he retired. His fruitful academic efforts continued for years and he taught medical students and residents and saved many newborn lives. Dr. Madani was the first neonatologist who graduated from Shiraz University of Medical Sciences and in 1990, this university was approved to accept neonatal residents.^2^

 One of Dr. Madani’s academic interests was supervision of the MD theses in neonatology. (Figure 1 shows an example.)

**Figure 1:**
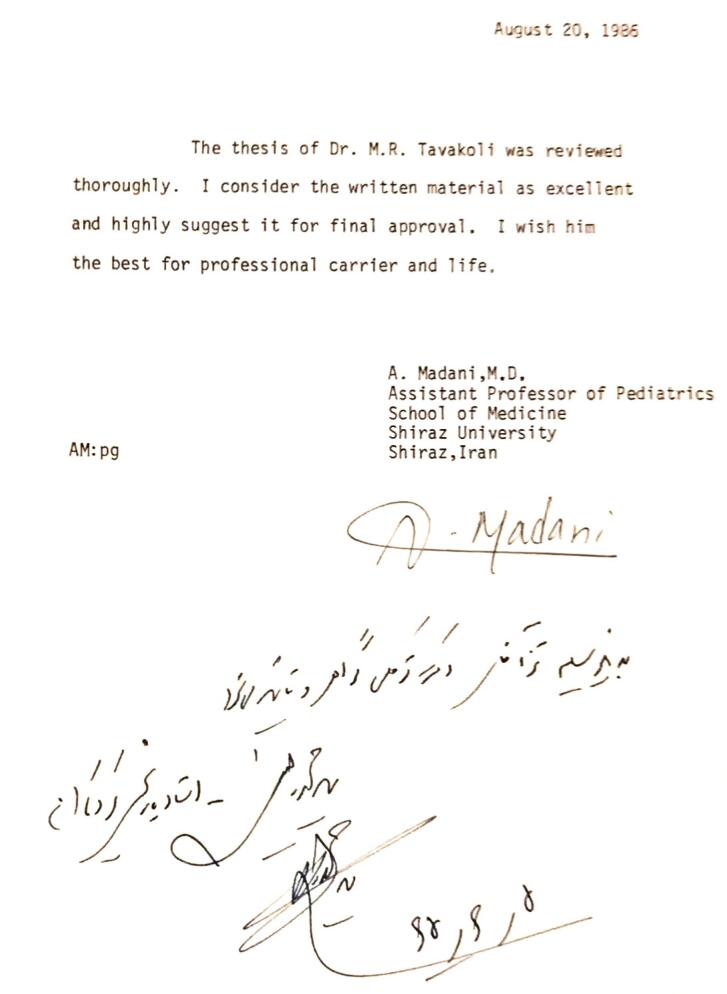
A Medical Thesis Supervised by Dr. Madani, 1986

 Dr. Madani loved neonatology and babies care and tried to improve their health and educate mothers on finding proper solution for their newborn’s daily problems (Figure 2). He wrote a book entitled “Newborns’ Special Care” for medical students. He was also a co-author alongside Professor Sedaghatian in a book named “Neonatal Emergencies” (Figure 3).

**Figure 2:**
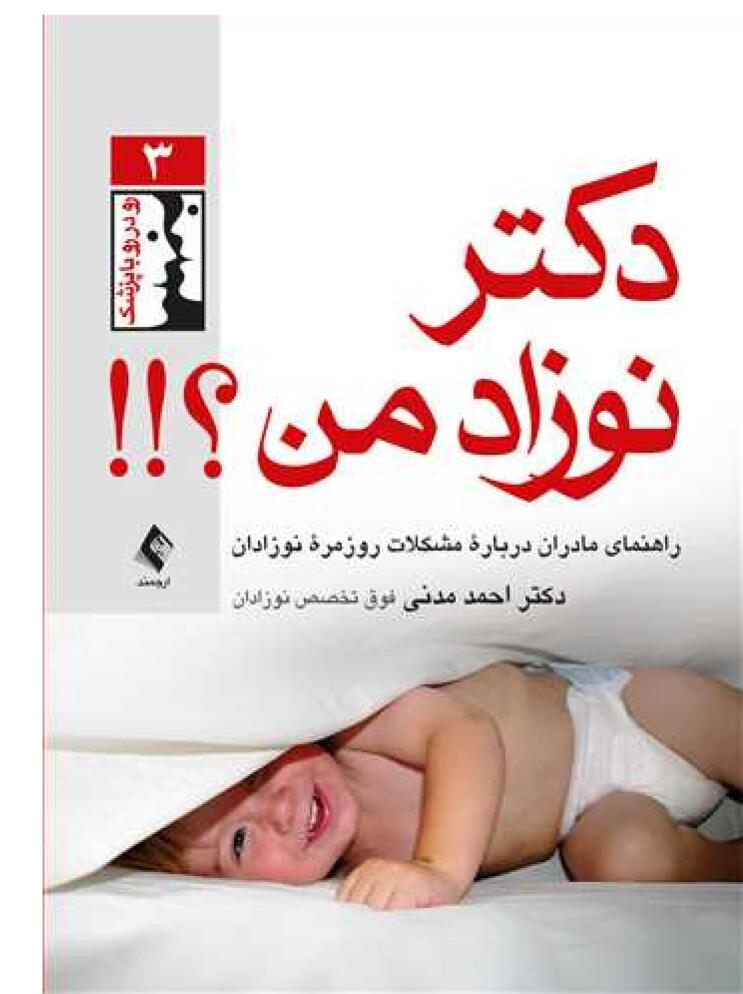
A Persian Guide for Mothers about Everyday Problems of the Babies, Authored by Dr. Madani

**Figure 3:**
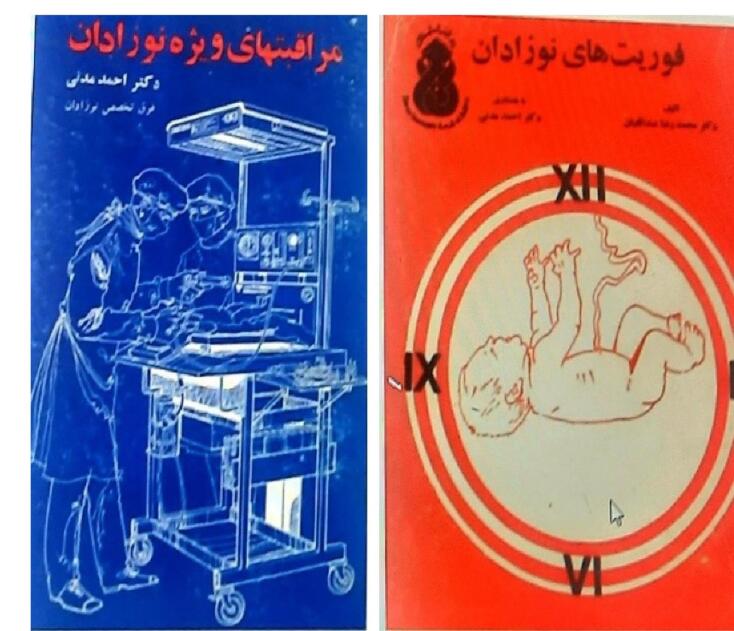
Two Persian Books “Neonatal Emergencies” and “Newborns’ Special Care”, Published by Shiraz University of Medical Sciences

 In addition to academic duties and treatment of his patients, he was highly interested in literature, music and history, especially history of medicine in Iran. He always appreciated the scientific efforts of the Iranian medical pioneers and wrote a comprehensive article regarding the development and pioneers of the department of pediatrics at Shiraz Medical School (Figure 4).^2^

**Figure 4:**
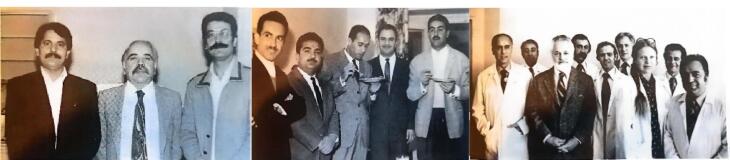
Faculty of the Shiraz School of Medicine at Pediatric Department.^2^ A) From left to right: Dr. Habibulla Javid, Dr. Mohammad Reza Sedaghatian and Dr. Madani.B) from left, second to third, Dr. Reza Amin, Dr. Reza Gharib, and Dr. S. Kavousi.C) Faculty of pediatric department at Shiraz School of Medicine in 1978

 In addition to academic duties, he was a prolific and creative author who wrote three interesting literary and cultural Persian books (Figure 5). He wrote that when he was a medical student in Isfahan, he and his friends were invited to the house of Dr. Parviz Dabiri, the professor and founder of the pathology department. Then, he added, “That night, Professor Dabiri after asking about our musical and artistic interests, he planned a program for us”. Dr. Madani then added, “After that, our music sessions with Professor Dabiri continued until one night at his suggestion, the core of the idea of establishing the “Isfahan Philharmonic Society” was conceived”.

**Figure 5:**
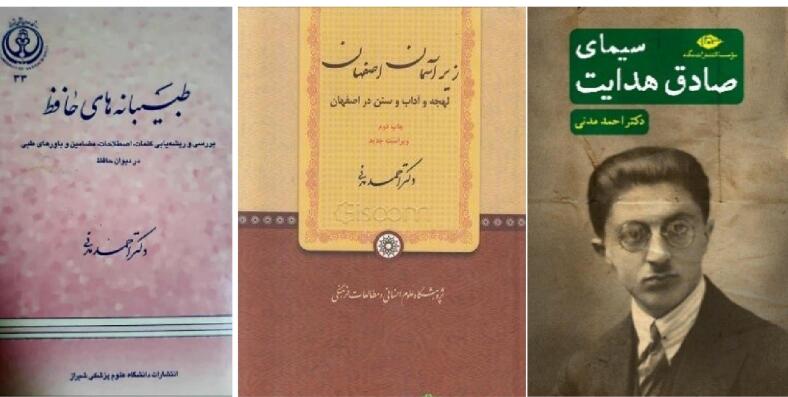
Three Literary Works of Dr. Madani.: from left, The medical beliefs of the great Iranian poet, Hafez, Under the sky of Isfahan and Simaye Sadegh Hedayat

 In addition, Dr. Madani was very loving of poetry, literature and humor and published articles and books in this fields. In his first Persian book, published in 2000, Dr. Madani, includes the medical beliefs of the great Iranian poet, Hafez (ca. 1315–1390) in his poetry book by investigating the roots of medical terms and themes. The first part contains three articles in which the behavior and character of doctors and their behavior with the patients from Hafez’s viewpoints are described. In the second part, in seven articles, Hafez’s viewpoints on the eye are described and analyzed. In the following parts, in several articles, folk beliefs of the moon and full moon, diseases, hospitals and elements as well as medicinal arrays, medicinal aromas and therapeutic methods are described in the poetry book of Hafez.^3^ The famous contemporary scholar of Hafez, Bahauddin Khormshahi, says about this book of Dr. Madani, “The valuable book of Hafez’s medical points is considered as one of the outstanding works in Hafez studies. It is probably the first book that has been written as a study about the medical themes in the poems of a poet and thinker”.^4^

 Another valuable work by Dr. Madani is called “Under the sky of Isfahan (dialect, customs and traditions in Isfahan)” which is actually a comprehensive cultural examination of the Isfahani dialect and its use.^5^ This book was published by “Research Institute of Humanities and Cultural Studies” in 950 pages and its first edition was published in 2013. In the first part of the book, the author reviews his childhood memories, which is actually a review of the past culture and life of the people of Isfahan at that time. Its prose is lovely and sweet. The last literary work written by Dr. Madani is called “Simaye Sadegh Hedayat”.

 The memories of professor Madani are unforgettable. The author was fortunate to spend some time, years ago in the neonatal department of Dr. Madani Hafez Hospital in Shiraz during his internship. He was always present at the bedside of hospitalized babies with a smiling and loving face, and visited the patients with commendable care and passion, and kindly answered our questions. Therefore, we considered him as a compassionate professor, in such a way that after graduation, our friendship continued. When I was editing the book “Physicians in the realm of culture and art” in 2009, I asked him to send me his biography, photos and examples of his literary works and he graciously accepted.^1^ Later, when compiling the first volume of the five-volume set of “A Collection of Essays on the History of Shiraz Medical School”, he wrote a comprehensive article on the history of the departments of pediatrics and neonatology of Shiraz Medical School and sent the photos given to him by the late Professor Reza Gharib. In addition, in 2013, when we were preparing the book “Departed Companion, In Memory of Professor Parviz Dabiri”, which I co-authored with Dr. Shahriar Dabiri, Dr. Madani wrote his memories of Professor Parviz Dabiri which were included in the book.^2^

 Finally, in the morning of April 17, 2024, a funeral ceremony was held with a significant number of professors, assistants and colleagues of Dr. Madani in the Shiraz Namazi Hospital (Figure 6) and then, his body was transferred to Isfahan and buried in Imamzadeh Mir Emad.

**Figure 6:**
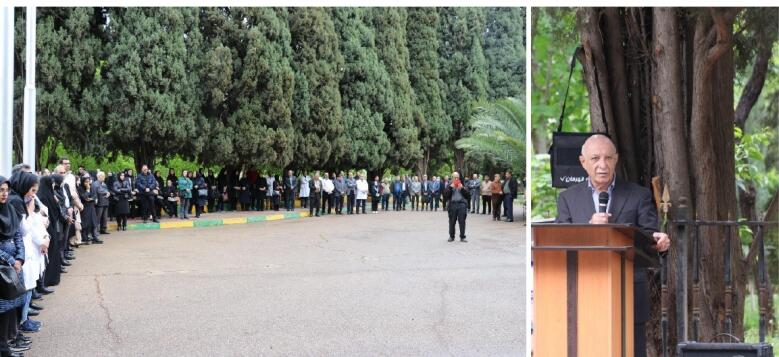
Dr. Mahmoud Haghighat, the President of the Fars Pediatric Society, Delivering a Speech at the Funeral. Memorial Ceremony of Dr. Madani at Namazi Hospital in Shiraz (Courtesy of Ms. Frough Ayat, Public Relation of Namazi Hospital in Shiraz)

## References

[1] Azizi MH. Pezeshkan dar Ghalamro Farhang va Honar (Physicians in the Realm of Culture and Art). Tehran: Mirmah Publications; 2009. p. 262-9. [Persian].

[2] Madani A. The history of pediatric and neonatology departments, in: A Collection of Essays on the History of Shiraz Medical School, In the Honor of Professor Faramarz Ismail- Beigi, by Dr. Mohammad Hossein Azizi, Mirmah Publication, Tehran, 2012:136-152.

[3] Madani A. Hafez’s Medical Points. 1st ed. Shiraz University Publication Center; 2000. p. 9-10, 42-4.3. [Persian].

[4] Khormshahi, Bahauddin: Bukhara Monthly Cultural Journal, [in Persian], p. 358, number 18, 2001.

[5] Madani A. Under the Sky of Isfahan (Dialect, Customs and Traditions in Isfahan). Research Institute of Humanities and Cultural Studies. 2011. [Persian].

